# And Justice for All? There Is More to the Interoperability of Contact Tracing Apps Than Legal Barriers. Comment on “COVID-19 Contact Tracing Apps: A Technologic Tower of Babel and the Gap for International Pandemic Control”

**DOI:** 10.2196/26218

**Published:** 2021-05-26

**Authors:** Rik Crutzen

**Affiliations:** 1 Department of Health Promotion Care and Public Health Research Institute Maastricht University Maastricht Netherlands

**Keywords:** COVID-19, contact tracing, data protection, privacy, interoperability, global health, public health

Du and colleagues [1] have conducted a thorough analysis of legal barriers for realizing the interoperability of contact tracing apps and emphasize the need for developing coordinated solutions to promote safe international travel and global control of the COVID-19 pandemic. Possible options for destroying the technological Tower of Babel are proposed that, in my opinion, are not necessarily needed due to legal barriers and warrant a broader reflection.

First, Du et al [1] note that the region-based development of contact tracing apps, along with the data protection laws used in different countries and regions, has resulted in a disconnection between contact tracing apps. A plea is made for a broader consensus in the international community. Within the European Union (EU), interoperability guidelines for contact tracing apps were already adopted by consensus by the eHealth Network in May 2020 [2]. So far, 22 countries make use of a contact tracing app that is in principle interoperable through a federation gateway [3].

Du et al [1] are partly right in stating that this will only solve the problem within the EU, partly because the GDPR (General Data Protection Regulation) imposes strict limitations on countries outside the EU to process data transfers. In short, other countries need to meet the high standards set by the GDPR in terms of personal data protection (for *all* individuals within the EU regardless of citizenship, including refugees and tourists). In other words, the GDPR does not rule out data transfer *in principle*, but sets high standards [4]. This because the right to protection of personal data is part of the Charter of Fundamental Rights of the EU [5]. Article 8 of the Charter clearly indicates that “everyone has the right to protection of personal data concerning him or her.”

Second, Du et al [1] offer a bold proposal: a common contact tracing app that is accepted by all countries and made mandatory for international travelers. Technically, the Google-Apple API (application programming interface) allows governments to work on developing their own contract tracing apps that are interoperable. Although this is not the same as the proposed app, it is a common interface that can be used across countries.

More importantly, even if all legal barriers are addressed when adopting such an interface on a global scale, making a contact tracing app mandatory is too bold a proposal. This is not (only) a legal concern, but (even more so) an ethical concern. Morley and colleagues [6] have synthesized 16 questions concerning factors that should be satisfied in order for a contact tracing app to be ethical. An important factor dictates that downloading and installing such an app should be optional. People should also not be penalized for noncompliance. Morley et al [6] acknowledge that these questions are likely to generate disagreement in terms of satisfying and prioritizing factors.

So, I agree with Du et al [1] on the need for developing coordinated solutions, but this should not only be focused on addressing legal barriers. Instead, such solutions should make optimal use of readily available technology and take ethical concerns seriously. In a raging pandemic, it might be alluring to take an everything-but-the-kitchen-sink approach and focus solely on controlling COVID-19. However, especially in a crisis, this “is dangerous when it ignores the real costs, including serious and long-lasting harms to fundamental rights and freedoms” [6]. This is what makes them fundamental.

## Abbreviations

API: application programming interface
EU: European Union
GDPR: General Data Protection Regulation

## References

[1] Du L, Raposo VL, Wang M (2020). COVID-19 Contact Tracing Apps: A Technologic Tower of Babel and the Gap for International Pandemic Control. JMIR Mhealth Uhealth.

[2] (2020). Interoperability guidelines for approved contact tracing mobile applications in the EU. eHealth Network.

[3] (2020). Explanatory memorandum on temporary law notification application Covid-19. Dutch Ministry of Health, Welfare and Sport.

[4] (2016). Regulation (EU)/679 of the European Parliament and of the Council of 27 April on the protection of natural persons with regard to the processing of personal data and on the free movement of such data, and repealing Directive 95/46/EC. European Parliament and Council.

[5] (2012). Charter of Fundamental Rights of the European Union. European Union.

[6] Morley Jessica, Cowls Josh, Taddeo Mariarosaria, Floridi Luciano (2020). Ethical guidelines for COVID-19 tracing apps. Nature.

